# Crystal structure of 2,6-di­chloro-4-nitro­pyridine *N*-oxide

**DOI:** 10.1107/S2056989015017387

**Published:** 2015-09-26

**Authors:** Andrew M. Prichard, Will E. Lynch, Clifford W. Padgett

**Affiliations:** a Armstrong State University, 11935 Abercorn St., Savanah, GA 31419, USA

**Keywords:** crystal structure, pyridine *N*-oxide, herringbone pattern

## Abstract

In the title compound, C_5_H_2_Cl_2_N_2_O_3_, the nitro group is essentially coplanar with the aromatic ring, with a twist angle of 4.00 (6)° and a fold angle of 2.28 (17)°. The crystal structure exhibits a herringbone pattern with the zigzag running along the *b* axis. The herringbone layer-to-layer distance is 3.0075 (15) Å, with a shift of 5.150 (4) Å. Neighboring mol­ecules are tilted at a 57.83 (4)° (ring-to-ring) angle with each other. The nitro group on one mol­ecule points to the *N*-oxide group on the neighboring one, with an inter­molecular O⋯N(nitro) distance of 3.1725 (13) Å.

## Related literature   

For the synthesis of the title compound and related compounds, see: Rousseau & Robins (1965 ▸). For chemical inter­est in derivatives of pyridine *N*-oxide, including the ruthenium-catalyzed use of these compounds towards the epoxidation of olefins *via* an *N*-oxide coordinated Ru^IV^=O inter­mediate, see: Gross & Ini (1999 ▸).
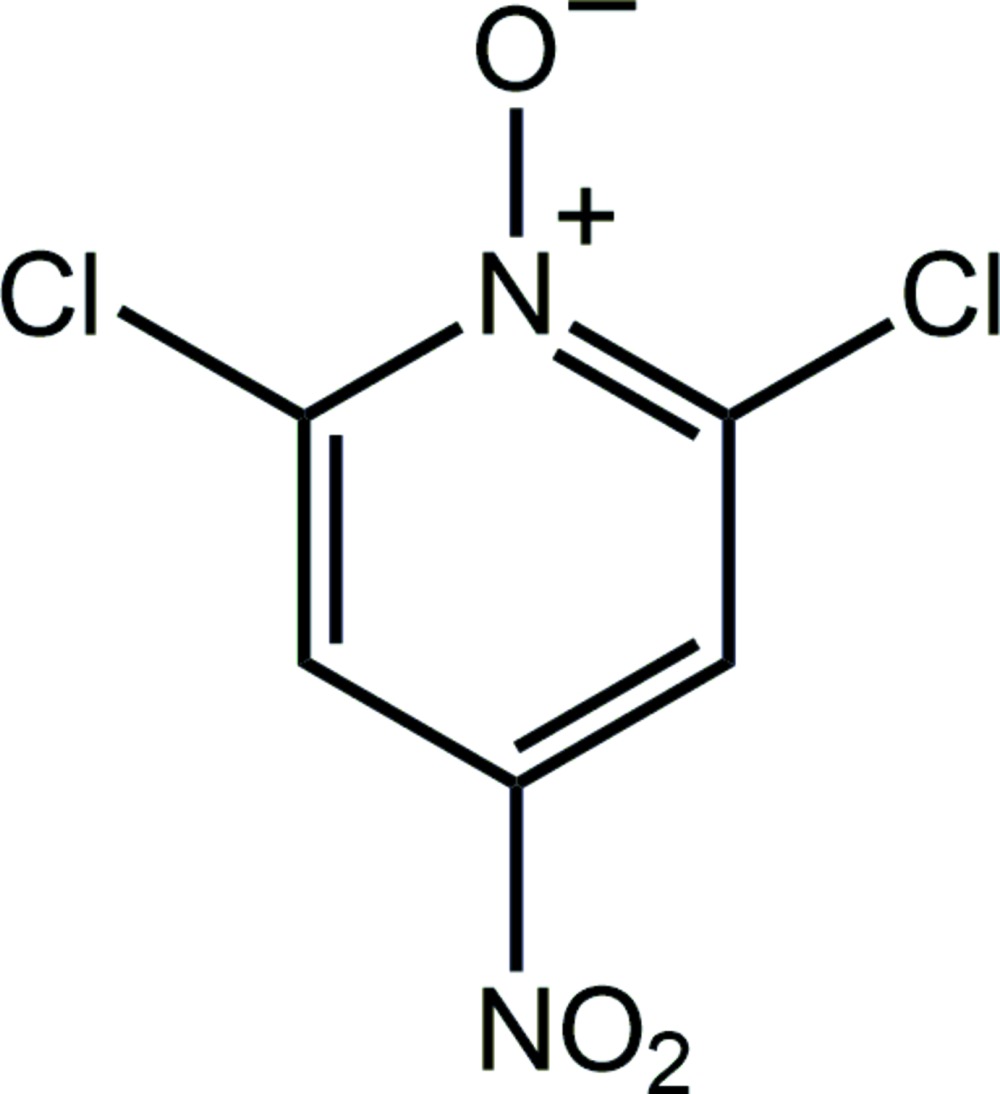


## Experimental   

### Crystal data  

C_5_H_2_Cl_2_N_2_O_3_
*M* *_r_* = 208.99
Orthorhombic, 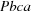

*a* = 5.964 (4) Å
*b* = 9.510 (6) Å
*c* = 26.192 (16) Å
*V* = 1485.5 (16) Å^3^
*Z* = 8
Mo *K*α radiation
μ = 0.84 mm^−1^
*T* = 173 K
0.6 × 0.2 × 0.1 mm

### Data collection  

Rigaku XtaLAB mini diffractometer
Absorption correction: multi-scan (*REQAB*; Rigaku, 1998 ▸) *T*_min_ = 0.709, *T*_max_ = 1.000
13695 measured reflections
1697 independent reflections
1512 reflections with *I* > 2σ(*I*)
*R*_int_ = 0.043

### Refinement  

*R*[*F* ^2^ > 2σ(*F* ^2^)] = 0.032
*wR*(*F* ^2^) = 0.078
*S* = 1.09
1697 reflections
109 parameters
H-atom parameters constrained
Δρ_max_ = 0.23 e Å^−3^
Δρ_min_ = −0.34 e Å^−3^

### 

Data collection: *CrystalClear-SM Expert* (Rigaku, 2011 ▸); cell refinement: *CrystalClear-SM Expert*; data reduction: *CrystalClear-SM Expert*; program(s) used to solve structure: SHELXT (Sheldrick, 2015*a* ▸); program(s) used to refine structure: *SHELXL2014* (Sheldrick, 2015*b* ▸); molecular graphics: *OLEX2* (Dolomanov *et al.*, 2009 ▸); software used to prepare material for publication: *OLEX2*.

## Supplementary Material

Crystal structure: contains datablock(s) I. DOI: 10.1107/S2056989015017387/tk5385sup1.cif

Structure factors: contains datablock(s) I. DOI: 10.1107/S2056989015017387/tk5385Isup2.hkl

Supporting information file. DOI: 10.1107/S2056989015017387/tk5385Isup3.cml

**Fig. 1.**
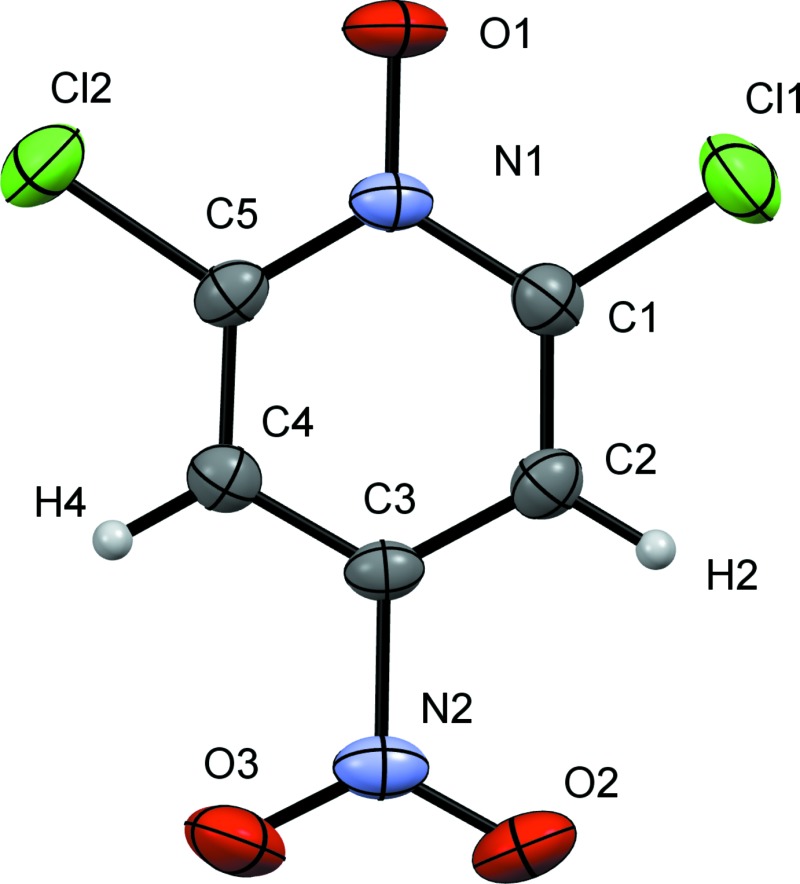
A view of the molecular structure of the title compound, with atom labelling. Displacement ellipsoids are drawn at the 50% probability level.

. DOI: 10.1107/S2056989015017387/tk5385fig1.tif
A view of the mol­ecular structure of the title compound, with atom labelling. Displacement ellipsoids are drawn at the 50% probability level.

CCDC reference: 1425488

Additional supporting information:  crystallographic information; 3D view; checkCIF report

## supplementary crystallographic information

### S1. Synthesis and crystallization

2,6-Di­chloro-4-nitro­pyridine *N*-oxide was purchased from Sigma-Aldrich and 0.10 g was dissolved in approximately 50 mL of methanol. Diffraction quality crystals were obtained by slow evaporation of the solvent.

### S2. Refinement

Carbon-bound H-atoms were placed in calculated positions (C—H = 0.95 Å) and were included in the refinement in the riding model approximation, with *U*_iso_(H) set to 1.2*U*_equiv_(C).

### Crystal data

**Table:**

C_5_H_2_Cl_2_N_2_O_3_	*D*_x_ = 1.869 Mg m^−^^3^
*M**_r_* = 208.99	Mo *K*α radiation, λ = 0.71073 Å
Orthorhombic, *P**b**c**a*	Cell parameters from 3422 reflections
*a* = 5.964 (4) Å	θ = 2.1–27.5°
*b* = 9.510 (6) Å	µ = 0.84 mm^−^^1^
*c* = 26.192 (16) Å	*T* = 173 K
*V* = 1485.5 (16) Å^3^	Prism, colorless
*Z* = 8	0.6 × 0.2 × 0.1 mm
*F*(000) = 832	

### Data collection

**Table:**

Rigaku XtaLAB mini diffractometer	1697 independent reflections
Radiation source: Sealed Tube	1512 reflections with *I* > 2σ(*I*)
Graphite Monochromator monochromator	*R*_int_ = 0.043
Detector resolution: 13.6612 pixels mm^-1^	θ_max_ = 27.5°, θ_min_ = 3.1°
profile data from ω scans	*h* = −7→7
Absorption correction: multi-scan (*REQAB*; Rigaku, 1998)	*k* = −12→12
*T*_min_ = 0.709, *T*_max_ = 1.000	*l* = −33→34
13695 measured reflections	

### Refinement

**Table:**

Refinement on *F*^2^	Primary atom site location: structure-invariant direct methods
Least-squares matrix: full	Hydrogen site location: inferred from neighbouring sites
*R*[*F*^2^ > 2σ(*F*^2^)] = 0.032	H-atom parameters constrained
*wR*(*F*^2^) = 0.078	*w* = 1/[σ^2^(*F*_o_^2^) + (0.026*P*)^2^ + 0.628*P*] where *P* = (*F*_o_^2^ + 2*F*_c_^2^)/3
*S* = 1.09	(Δ/σ)_max_ = 0.001
1697 reflections	Δρ_max_ = 0.23 e Å^−^^3^
109 parameters	Δρ_min_ = −0.34 e Å^−^^3^
0 restraints	

### Special details

**Table:**

Geometry. All e.s.d.'s (except the e.s.d. in the dihedral angle between two l.s. planes) are estimated using the full covariance matrix. The cell e.s.d.'s are taken into account individually in the estimation of e.s.d.'s in distances, angles and torsion angles; correlations between e.s.d.'s in cell parameters are only used when they are defined by crystal symmetry. An approximate (isotropic) treatment of cell e.s.d.'s is used for estimating e.s.d.'s involving l.s. planes.

### Fractional atomic coordinates and isotropic or equivalent isotropic displacement parameters (Å^2^)

**Table:**

	*x*	*y*	*z*	*U*_iso_*/*U*_eq_	
Cl2	0.85784 (8)	0.45634 (5)	0.29466 (2)	0.04219 (16)	
Cl1	0.76404 (10)	0.58023 (6)	0.48666 (2)	0.05096 (18)	
O1	0.9698 (2)	0.47138 (13)	0.39871 (5)	0.0406 (3)	
O2	0.0755 (2)	0.79615 (14)	0.39133 (6)	0.0436 (3)	
O3	0.1322 (2)	0.76147 (15)	0.31036 (6)	0.0494 (4)	
N1	0.7830 (2)	0.53287 (14)	0.38874 (5)	0.0278 (3)	
N2	0.1835 (2)	0.74825 (15)	0.35543 (6)	0.0337 (3)	
C3	0.3887 (3)	0.67006 (16)	0.36721 (6)	0.0261 (3)	
C4	0.5076 (3)	0.60983 (16)	0.32781 (6)	0.0273 (3)	
H4	0.4563	0.6160	0.2935	0.033*	
C2	0.4594 (3)	0.66162 (17)	0.41700 (7)	0.0297 (4)	
H2	0.3734	0.7017	0.4439	0.036*	
C5	0.7030 (3)	0.54043 (16)	0.33971 (6)	0.0268 (3)	
C1	0.6574 (3)	0.59381 (17)	0.42689 (7)	0.0302 (4)	

### Atomic displacement parameters (Å^2^)

**Table:**

	*U*^11^	*U*^22^	*U*^33^	*U*^12^	*U*^13^	*U*^23^
Cl2	0.0471 (3)	0.0357 (3)	0.0438 (3)	0.0101 (2)	0.0179 (2)	0.00214 (18)
Cl1	0.0628 (4)	0.0529 (3)	0.0372 (3)	0.0068 (2)	−0.0209 (2)	−0.0034 (2)
O1	0.0271 (6)	0.0318 (7)	0.0630 (9)	0.0093 (5)	−0.0097 (6)	0.0019 (6)
O2	0.0319 (7)	0.0348 (7)	0.0639 (9)	0.0093 (6)	0.0106 (6)	0.0024 (6)
O3	0.0438 (8)	0.0493 (9)	0.0552 (9)	0.0111 (7)	−0.0184 (7)	0.0051 (7)
N1	0.0229 (7)	0.0207 (7)	0.0397 (8)	0.0013 (5)	−0.0042 (6)	0.0013 (6)
N2	0.0260 (7)	0.0242 (7)	0.0508 (9)	0.0009 (6)	−0.0035 (7)	0.0047 (6)
C3	0.0223 (7)	0.0202 (7)	0.0357 (9)	0.0005 (6)	−0.0006 (6)	0.0026 (6)
C4	0.0293 (8)	0.0223 (8)	0.0303 (8)	−0.0013 (6)	−0.0016 (7)	0.0021 (6)
C2	0.0325 (9)	0.0238 (8)	0.0330 (9)	0.0021 (7)	0.0018 (7)	−0.0019 (7)
C5	0.0281 (8)	0.0204 (8)	0.0319 (9)	0.0006 (6)	0.0045 (7)	0.0012 (6)
C1	0.0341 (9)	0.0255 (8)	0.0310 (9)	−0.0005 (7)	−0.0050 (7)	−0.0006 (6)

### Geometric parameters (Å, º)

**Table:**

Cl2—C5	1.6984 (18)	N2—C3	1.465 (2)
Cl1—C1	1.695 (2)	C3—C4	1.377 (2)
O1—N1	1.2847 (18)	C3—C2	1.373 (2)
O2—N2	1.228 (2)	C4—H4	0.9500
O3—N2	1.226 (2)	C4—C5	1.375 (2)
N1—C5	1.372 (2)	C2—H2	0.9500
N1—C1	1.377 (2)	C2—C1	1.370 (3)
			
O1—N1—C5	121.03 (14)	C5—C4—H4	121.1
O1—N1—C1	121.06 (15)	C3—C2—H2	120.9
C5—N1—C1	117.90 (14)	C1—C2—C3	118.13 (16)
O2—N2—C3	117.74 (15)	C1—C2—H2	120.9
O3—N2—O2	124.65 (16)	N1—C5—Cl2	115.89 (13)
O3—N2—C3	117.61 (15)	N1—C5—C4	122.15 (15)
C4—C3—N2	118.91 (15)	C4—C5—Cl2	121.96 (14)
C2—C3—N2	119.10 (15)	N1—C1—Cl1	115.73 (13)
C2—C3—C4	121.98 (16)	C2—C1—Cl1	122.26 (14)
C3—C4—H4	121.1	C2—C1—N1	122.01 (16)
C5—C4—C3	117.79 (16)		
