# Supplementary figures and images for: Crystal structure of 2,6-di­chloro-4-nitro­pyridine N-oxide
Source: Acta Crystallogr E Crystallogr Commun. 2015 Sep 26;71(Pt 10):o775. doi: 10.1107/S2056989015017387 (PMC4647420; doi:10.1107/S2056989015017387)

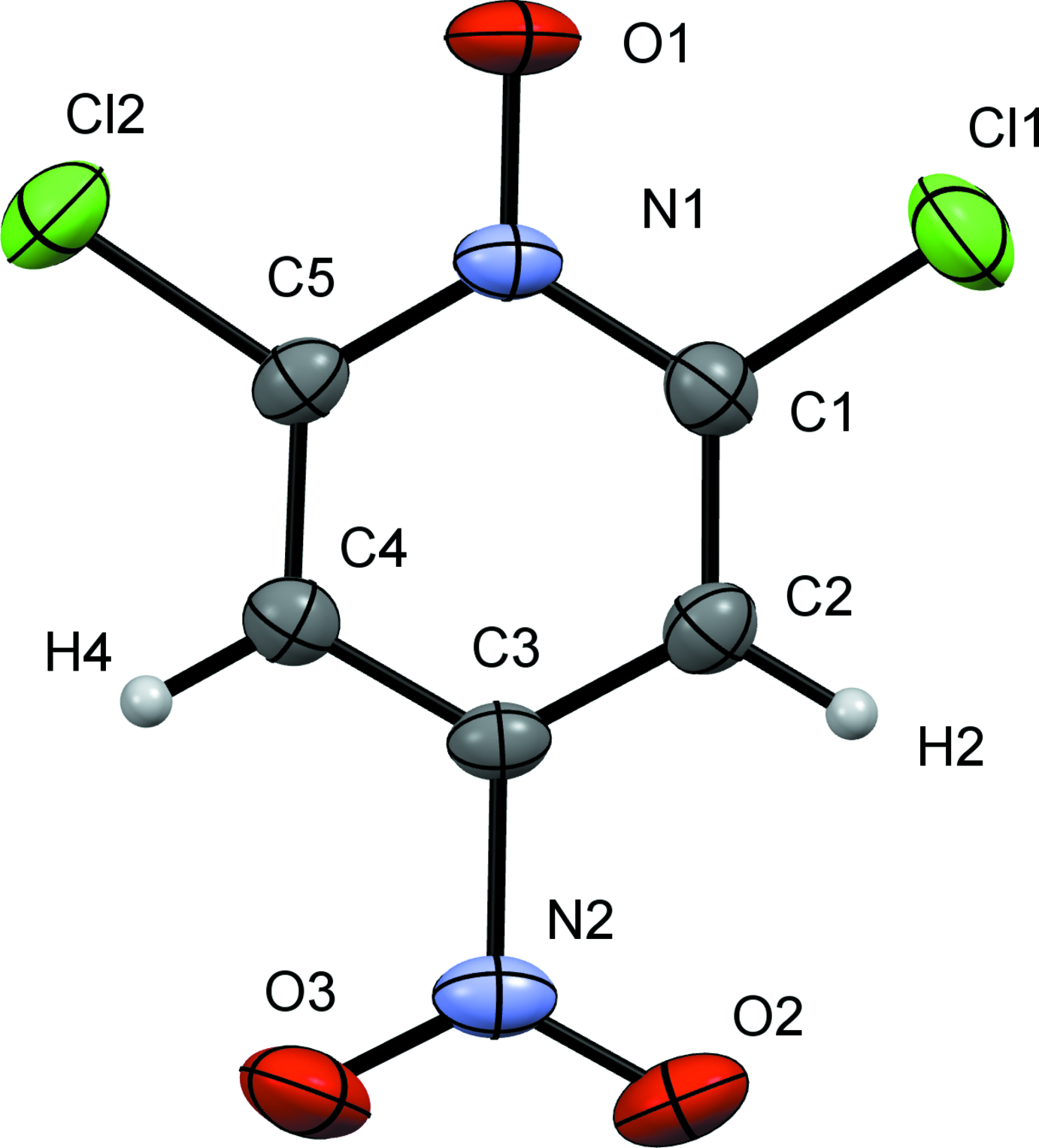

Supplement: Supplementary file 4 [file e-71-0o775-fig1.tif]
